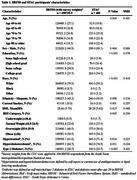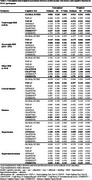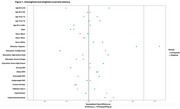# Cardiovascular Risk Factors and Cognitive Function ‐ Generalizing Results from a Clinical Cohort

**DOI:** 10.1002/alz70861_108622

**Published:** 2025-12-23

**Authors:** Janette Vazquez, Ashley LaRoche, Daniel MacCarthy, Roman A. Fernandez, Silvia Mejia‐Arango, Monica Goss, Hector A Trevino, Vanessa M. Young, Gabrielle Hromas, Amy Werry, Arash Salardini, Bruce R Leforce, Jeremy A. Tanner, A. Campbell Sullivan, Helen Hazuda, Jesus D Melgarejo, Gladys E. Maestre, Sudha Seshadri, Chen‐Pin Wang, Claudia L Satizabal

**Affiliations:** ^1^ Glenn Biggs Institute for Alzheimer's & Neurodegenerative Diseases, University of Texas Health Science Center, San Antonio, TX USA; ^2^ Institute of Neuroscience at the University of Texas Rio Grande Valley, Harlingen, TX USA

## Abstract

**Background:**

Generalizing dementia research findings from clinical studies to broader populations is challenging due to selective participation. This study evaluated associations between cardiovascular risk factors and cognitive function in the South Texas Alzheimer’s Disease Research Center cohort (STAC), leveraging 2023 Behavioral Risk Factor Surveillance System (BRFSS) data, a nationwide survey collecting health‐related factors, to project findings to South Texas older adults ‐ the target population.

**Method:**

Participants included 483 dementia‐free adults aged ³60 from STAC (mean age 73.7±7.3) and a representative sample of adults aged ³60 from the 2023 BRFSS for the South Texas Metropolitan/Micropolitan areas (Table 1). Variables were harmonized, and STAC participation probability and generalizability weights calculated to align covariate distributions with the target population. Covariate alignment pre‐ and post‐weighting was assessed using standardized mean differences (Figure 1). Unweighted and weighted regression models, adjusted for age, sex, education, race, and ethnicity, evaluated associations between cardiovascular risk factors (BMI, hypercholesterolemia, hypertension, diabetes, smoking) and cognitive function (verbal fluency, executive function, attention, immediate/delayed memory, visuoconstructional ability, global cognition) for STAC and the targeted population, respectively.

**Results:**

STAC participants were older, more educated, and more likely to self‐report as White than the target population. Unweighted analyses suggested hypertension was associated with lower verbal fluency (b=‐1.3±0.59, *p* =0.03), and BMI³30 (vs BMI 18.5‐24.9) with better immediate and delayed verbal memory (b=2.56±0.89, *p* <0.01; b=2.06±1.01, *p* =0.04) in STAC ‐ associations not present in the target population after weighting. Conversely, weighted analyses in the target population revealed associations between hypercholesterolemia and poorer visuoconstructional memory (b=‐1.63±0.64, *p* =0.01), BMI 25‐29.9 and reduced executive function (b=‐0.19±0.09, *p* =0.04), diabetes and better visuoconstructional performance (b=0.125±0.06, *p* =0.03), and current smoking and decreased attention (b=‐0.30±0.13, *p* =0.02). Significant associations between BMI£18.4 and worse global cognition, executive function, and visuoconstructional performance, as well as BMI³30 and better visuoconstructional performance were found in both groups (Table 2).

**Conclusion:**

Our findings highlight participants’ characteristics differences between STAC and the target population. Generalizability weighting may be a valuable tool to improve external validity, however, challenges remain in identifying representative cohorts with high quality data that allow us to infer generalizability of studies prone to selection bias. Additional analysis stratifying by sex and race/ethnicity are underway.